# Factors associated with the use of mosquito bed nets: results from two cross-sectional household surveys in Zambézia Province, Mozambique

**DOI:** 10.1186/s12936-016-1250-5

**Published:** 2016-04-11

**Authors:** Troy D. Moon, Caleb B. Hayes, Meridith Blevins, Melanie L. Lopez, Ann F. Green, Lazaro González-Calvo, Omo Olupona, The Ogumaniha-SCIP Zambézia Consortium

**Affiliations:** Vanderbilt Institute for Global Health, 2525 West End Avenue, Suite 750, Nashville, TN 37203 USA; Friends in Global Health, Maputo, Mozambique; Department of Biostatistics, Vanderbilt University, Nashville, TN USA; World Vision US, Federal Way, WA USA; World Vision International, Maputo, Mozambique; Department of Pediatrics, Division of Infectious Diseases, Vanderbilt University, Nashville, TN USA

**Keywords:** Malaria, Prevention, Long-lasting insecticide treated bed nets, Mozambique

## Abstract

**Background:**

Malaria remains a major threat to some 3.2 billion persons globally. Malaria contributes heavily to the overall disease burden in Mozambique and is considered endemic. A cornerstone of Mozambique’s vector control strategy has been to strive for universal coverage of insecticide-treated nets (ITN).

**Methods:**

The study is a population-based cross-sectional survey of female heads-of-household in Zambézia Province, Mozambique conducted during August–September, 2010 and April–May, 2014. Analyses accounted for a stratified two-stage cluster sample design. Outcomes of interest included sleeping under a mosquito net during the previous night. Descriptive statistics were calculated for three oversampled districts and for the entire province. Multivariable logistic regression analysis was used to estimate factors associated with both changes over time and increased mosquito bed net usage.

**Results:**

Of the 3916 households interviewed in 2010 and 3906 households in 2014, 64.3 % were in possession of at least one mosquito bed net. A higher proportion of households in Namacurra (90 %) reported possession of a mosquito net, compared to Alto Molócuè (77 %) and Morrumbala (34 %), respectively in 2014. Of pregnant respondents, 58.6 % reported sleeping under a mosquito net the previous night in 2010 compared to 68.4 % in 2014. Fifty percent of children 0–59 months slept under a mosquito net the previous night in 2010 compared to 60 % in 2014. Factors associated with use of a mosquito net for female head-of-household respondents were higher education, understanding Portuguese, larger household size, having electricity in the household, and larger household monthly income. As travel time to a health facility increased (per 1 h), respondents had 13 % lower odds of sleeping under a mosquito net (OR 0.87; 95 % CI 0.74–1.01, p = 0.07). Pregnant women in 2014 had a 2.4 times higher odds of sleeping under a bed net if they lived in Namacurra compared to Alto Molócuè (95 % CI 0.91–6.32, p = 0.002 for district). Higher maternal education, living in Namacurra, and acquisition of mosquito bed nets were associated with a child 0–59 months reporting sleeping under the net in the previous night in 2014.

**Conclusions:**

Intensified focus on the poorest, least educated, and most distant from health services is needed to improve equity of ITN availability and usage. Additionally, while some districts have already surpassed goals in terms of coverage and utilization of ITN, renewed emphasis should be placed on bringing all geographic regions of the province closer to meeting these targets.

## Background

Malaria remains a major threat to some 3.2 billion persons globally. According to the World Health Organization (WHO), 214 million cases of malaria occurred in 2015, leading to 438,000 deaths. Eighty-eight percent of these deaths occurred in the Africa region [1]. Malaria is one of the two leading causes of death in Mozambique, accounting for 29 % of all deaths in 2008 [2]. In 2011, malaria was reported as the leading cause of death in children under 5 years [3, 4]. Contributing heavily to the overall disease burden in Mozambique, malaria is considered endemic throughout the country [5]. Transmission occurs year round but with seasonal peaks during and directly after the rainy season, typically from December to April [5]. While Mozambique´s entire population of 26 million is considered at risk for malaria, regional prevalence can be quite different across the country, ranging from 1.5 % in Maputo in the south to 54.8 % in Zambézia Province in the north in 2011. Additionally, a threefold higher prevalence has been reported in rural areas compared to urban [3, 6].

Mozambique´s National Malaria Control Programme is responsible for developing policy, planning, and coordinating all malaria control activities within the country. In recent years, major investments have been made through programmes such as the United States President’s Malaria Initiative (PMI) and the Global Fund to Fight AIDS, Tuberculosis, and Malaria (Global Fund). Mozambique´s National Malaria Prevention and Control Strategic Plan: 2012–2016 continues to focus heavily on four proven and highly effective prevention and treatment strategies: insecticide-treated mosquito nets (ITNs), indoor residual spraying (IRS), diagnosis and treatment with artemisinin-based combination therapy (ACT), and intermittent preventive treatment of pregnant women (IPTp) [5].

For several years, a cornerstone of Mozambique’s vector control strategy has been to strive for universal coverage of ITNs [6]. Coverage and mosquito net usage have been slowly increasing amongst key target groups. For example, nationwide from 2007 to 2015 the proportion of children under 5 years of age using an ITN in the previous night increased from 7 to 36 % [5]. Surveillance surveys conducted as part of the Malaria Decision Support System Project in Zambézia Province, a high malaria prevalence region, reported an increase of ITN usage in children under 5 years from 29.8 % in 2006 to 34.3 % in 2009 [7]. Despite these reported increases, the country has not yet been able to report a consistent and sustained decline in malaria incidence [3–5, 8].

Current ITN distribution strategies prioritize extremely vulnerable populations and strive for 80 % coverage of young children and pregnant women [4, 5]. Evidence shows that further protective effects can be gained when sufficient portions of all individuals at risk consistently utilize mosquito nets. As such, coverage of entire populations will be required to achieve large reductions in malaria burden in endemic countries such as Mozambique [9]. However, ownership of nets does not always translate to proper utilization [10–12]. In 2009, Mozambique officially adopted a policy of universal coverage, defined as one ITN for every two persons [5]. Determining the best strategies for attaining and maintaining this universal coverage remains uncertain [13]. Challenges include the need for establishing appropriate algorithms for determining the number of mosquito nets needed for distribution [13]; promoting proper use and replacement of old and torn nets [14, 15]; as well as understanding the local social norms and other determinants that contribute to increased uptake and utilization [16–18].

Set against this backdrop, the *Ogumaniha* project began implementation in Zambézia Province, Mozambique in late 2009. The project is funded by the US Government under the United States Agency for International Development (USAID) Strengthening Communities through Integrated Programming (SCIP) award and implemented by a consortium of partners led by World Vision. In the local Echuabo language, *Ogumaniha* means “united/integrated for a common purpose”. The overarching goal of this 5-year project (currently extended into a 6th year) is to improve the health and livelihoods of women, children and families in Zambézia Province by pursuing an integrated, innovative, and sustainable community-based programme supporting cross-sector integration of USAID’s development actions in the province.

In order to achieve the above objectives, the Ogumaniha consortium structured its interventions through a mixture of training and capacity building of local community volunteer groups called community health committee’s (CHC), as well as direct implementation of activities by consortium partners. CHC’s are networks of volunteers within a community usually consisting of 20–30 volunteers. These volunteers are then divided into thematic areas of community support such as nutrition; maternal health/family planning/reproductive health; home based care; water and sanitation; home visits and active case finding for persons living with HIV; child health; local economic development; and malaria education and prevention. Ogumaniha’s malaria related activities primarily focused on community-based education messages and behaviour change communications promoting improved health-seeking behaviour, as well as the active identification and referral of suspected malaria cases in the community to a health facility for testing and treatment. In addition, the Ogumaniha consortium assisted provincial supply chain systems for the monthly transport of ITN´s to the province’s 200+ health facilities in order to stock programmes supporting universal distribution within antenatal care clinics and for children under 5 years of age seen at the health facility.

The implementation of mass ITN campaigns for universal coverage began a slow roll-out across the country in 2010. In 2012, with support from the Global Fund and World Bank, 11 of 17 districts in Zambézia Province benefitted from mass distribution. In line with the goals of the National Malaria Control Strategic Plan 2012–2016, plans were established for a coordinated increase in universal ITN distribution with Global Fund, World Bank and PMI support targeting 90 % of districts nationwide by the end of calendar year 2014 [5]. The Ogumaniha consortium was not directly involved in universal mass ITN campaigns, though World Vision, the consortiums principle partner, began supporting mass ITN campaigns in Zambézia Province with Global Fund support in 2015.

Integral to Ogumaniha’s design is a strong monitoring system and project evaluation based on performance indicators agreed upon with USAID and the provincial government. To estimate changes over time, population-based household surveys were conducted at the project´s beginning and end [19]. This report is a review of malaria specific cross-sectional survey data collected at baseline (August and September 2010) and endline (April and May 2014), in order to assess mosquito bed net possession and factors associated with their use.

## Methods

### Sample design and data collection

**Fig. 1:**
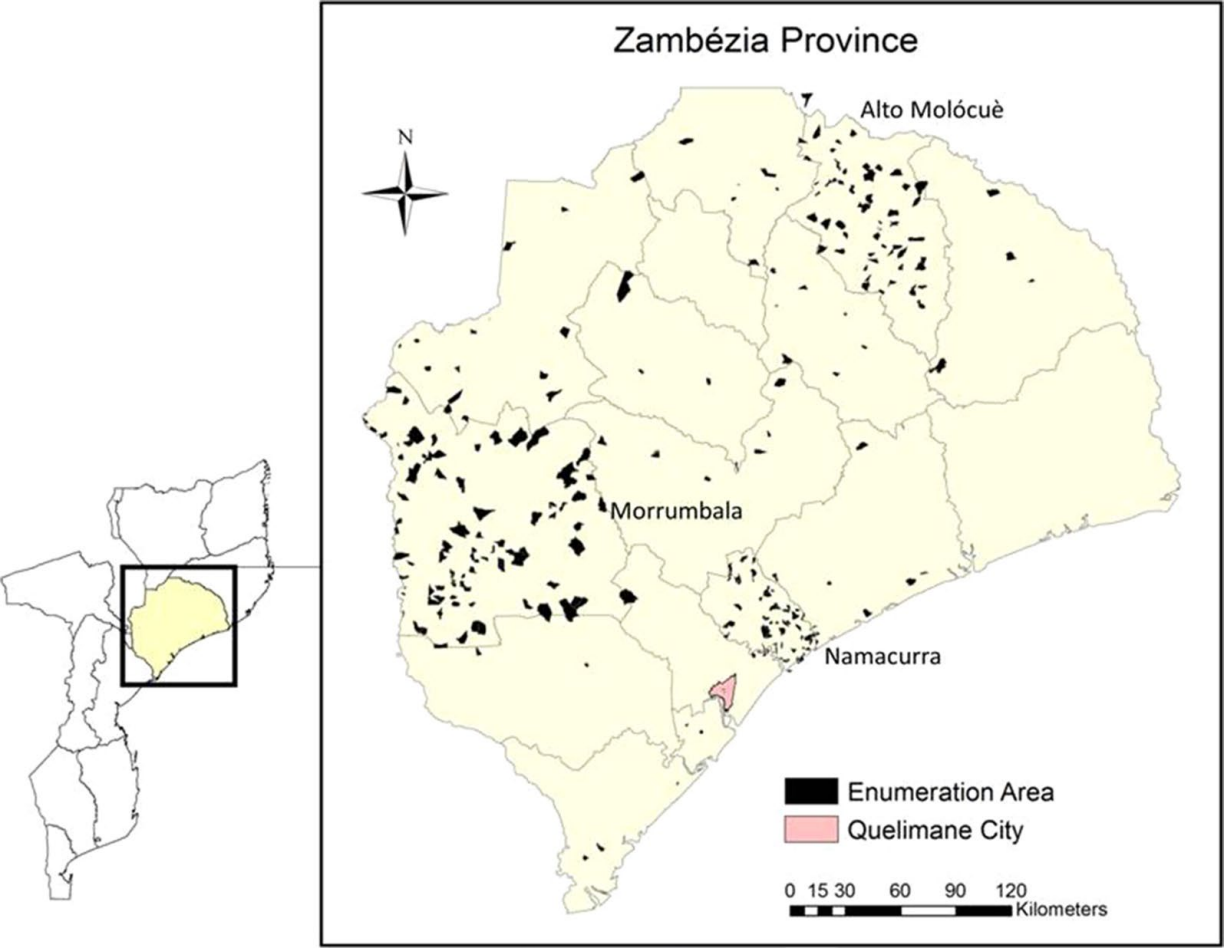
Map of Zambézia Province with enumeration areas surveyed. *Oversampled districts highlighted, Alto Molócuè, Morrumbala, and Namacurra Map credit: Charlotte Buehler; May 27 2015; Vanderbilt Institute for Global Health; Projection: WGS 1984 Web Mercator Auxiliary Sphere

The design and implementation of this study are detailed elsewhere [19–22]. Briefly, survey teams completed interviews in 262 enumeration areas (EA) across Zambézia Province. A large representative sample (201 EA) was obtained from three diverse districts (Alto Molócuè, Namacurra, and Morrumbala) in order to increase the precision of survey results while minimizing costs. To further maintain a degree of generalizability across the province, a sample of households were selected for interview throughout the remaining 14 districts (Fig. 1).

At both baseline and endline the same questionnaire was utilized. While survey responses were not collected from the same households in both surveys, the same sampling methodology was utilized with interviewers returning to the same EAs as in baseline. The Ogumaniha survey tool collects information on over 500 variables in eight dimensions and was developed by a multi-disciplinary team of researchers from Vanderbilt University and the University Eduardo Mondlane in Maputo. The survey designers borrowed many questions and scales deemed appropriate from previous national surveys in Mozambique and other international surveys such as the Demographic Health Survey (DHS) and Multiple Indicator Cluster Survey (MICS). Survey questions covered household demographics; economic status; health knowledge, attitudes and practices; access to health and malaria-related services and products; access to improved water and sanitation; nutritional intake; and others.

In both surveys, fourteen mobile teams consisting of a team leader and four interviewers administered the survey face-to-face using mobile phones with an electronic questionnaire installed for data collection. Local authorities were notified prior to the arrival of the survey team. From topographic maps, the survey teams divided the EAs into four quadrants. Starting in the center of the assigned quadrant, interviewers selected a direction, then chose the first household in that direction (i.e. starting point), and approached the nearest four households for interview. Female interviewers conducted the survey with female heads-of-household, defined as the only or principal wife of the immediate family of the household. The female head-of-household was chosen as it was felt she was the most likely person to be familiar with the health and care taking of the entire family. In polygamous families, the eldest wife was selected. Interviewers were trained to conduct interviews in Portuguese or in one of the five predominant local languages. Household surveys were conducted at the end of rainy season, representing the peak malaria transmission period for that year.

### Statistical analysis

All analyses accounted for a stratified two-stage cluster sample design. The three outcomes of interest included whether the female heads-of-household, pregnant female heads-of-household, or children aged 0–59 months slept under a mosquito bed net during the previous night. Descriptive statistics were calculated for three oversampled districts and for the entire province. Continuous variables were reported as weighted estimates of median (interquartile range [IQR]) and categorical variables were reported as weighted percentages, with each observation being weighted by the inverse of the household or child sampling probability. Multivariable logistic regression analysis with robust covariance estimation to account for clustering was used to estimate factors associated with bed net usage for the three groups of interest. Only households from the oversampled districts were included in the regressions. Covariates were identified a priori and they included: age, education, Portuguese understanding, household size, district, whether all bed nets were donations, whether all bed nets were purchased, monthly income, travel time to health facility, household electricity, bed net distribution at current pregnancy (pregnant group only), and recent fever in child (child group only). Family income was reported in *meticais (MZN)* (1USD ≈ 36MZN in August 2010 and 1USD ≈ 31MZN in April 2014). If there was evidence of non-linearity (Wald test p < 0.10), continuous variables were modeled using restricted cubic splines [23, 24]. Missing values of covariates were accounted for using multiple imputation techniques. R-software 3.2.2 was used for analyses.

### Ethical considerations

Participation in the household surveys was completely voluntary, no incentive was provided for participation. At enrollment written informed consent was obtained. Approvals for study implementation were obtained at the national level from the National Directorate for Public Health of the Ministry of Health (*Direcção Nacional de Saúde Publica*) and at the Provincial level from the Provincial Health Directorate of Zambézia Province (*Direcção Provincial de Saúde*-*Zambézia*). Survey protocol, questionnaires, and informed consent documents were approved by both the Inter-institutional Committee for Bioethics in Health-Zambézia (*Comité Inter*-*institucional de Bioética em Saude*-*Zambézia*) and the Vanderbilt University Institutional Review Board (IRB).

## Results

A total of 3916 surveys at baseline (2010) and 3906 surveys at endline (2014) were completed in 262 EAs across 14 districts in Zambézia Province. Of these, 7628 (97.5 %) surveys had sufficient data completed to be databased for analysis (Table 1). Table 2 summarizes demographic information for the female head-of-household respondents. The median age was 29 (IQR 23–37) in 2010 and 27 years (IQR 22–34) in 2014. Across both surveys, most women (>67 %) reported being married or in a common-law relationship. Province-wide, median years of formal education in 2010 was 2 years (IQR 0–4) compared to 3 years (IQR 0–5) in 2014. While very few women at endline (3.3 %) had a reported education beyond secondary school (10th grade), 25.4 % of women in Alto Molócuè and 14.8 % of women in Namacurra reported completing between 6 and 10 years of education, compared to Morrumbala, in which only 5 % of women reported completing more than 2 years of formal education. Median household size was five persons, with >75 % of households having a least one child under the age of 5 years. In both surveys, roughly 84 % of households interviewed reported a monthly household income of less than 1000 MZN per month (< $1 USD per day). Portuguese language understanding was limited with only 39 % reporting understanding at baseline and 43.1 % at endline. Most households (roughly 80 %) were located in rural areas, with only between 5 and 10 % of respondents reporting access to electricity in the home.

**Table 1:** Survey response

Survey collection status, n (%)	Baseline	Endline	Combined
Survey started	3916	3906	7822
Survey incomplete	164 (4.1 %)	14 (0.3 %)	178 (2.2 %)
Participant quits	1 (<0.1 %)	0 (0.0 %)	1 (<0.1 %)
Stopped for safety concern at location	2 (<0.1 %)	0 (0.0 %)	2 (<0.1 %)
Surveys analysed	3749 (95.7 %)	3892 (99.7 %)	7628 (97.5 %)

**Table 2:** Basic demographics: female head-of-household

	Baseline (n = 3749)	Endline (n = 3892)
Age of respondent	29 (23–37)	27 (22–34)
Marital status		
Single	17.4 %	28.6 %
Married/common law	74.0 %	67.9 %
Widowed	4.8 %	1.6 %
Divorced/separated	3.8 %	2.0 %
Years of education, median (IQR)	2 (0–4)	3 (0–5)
Education category		
0–5 years	86.9 %	79.7 %
6–10 years	12.1 %	17.1 %
>10 years	1.0 %	3.3 %
Household size, median (IQR)	5 (3–6)	5 (4–6)
Any child under age 5	76.7 %	86.9 %
Household income		
<1000 meticais per month	84.7 %	84.1 %
1000+ meticais per month	15.3 %	15.9 %
Head-of-household understands Portuguese	39.0 %	43.1 %
Primary language of household		
Cinyanja	15.0 %	14.3 %
Cisena	12.5 %	11.8 %
Echuabo	23.8 %	20.3 %
Elomwe	40.0 %	33.6 %
Emakhuwa	0.5 %	0.3 %
Nharinga	0.0 %	1.4 %
Portuguese	8.2 %	8.3 %
Urban/rural		
Rural	80.4 %	80.2 %
Urban	19.6 %	19.8 %
Household has electricity	4.9 %	10 %

Continuous variables are reported as weighted estimates of median (interquartile range), with each observation being weighted by the inverse of the household sampling probability

Categorical variables are reported as weighted percentages, with each observation being weighted by the inverse of the household sampling probability

Of households interviewed at baseline and endline, roughly 64 % were in possession of at least one mosquito bed net (Table 3). When analysed by the three oversampled districts, a much higher proportion of households in Namacurra (90 %) reported possessing a mosquito net, compared to Alto Molócuè (77 %) and Namacurra (34 %), respectively in 2014. When asked to respond to “*who normally sleeps under the mosquito net?”* roughly 50 % of respondents in both surveys reported “*everyone*”, however district specific differences existed. At endline, Alto Molócuè and Namacurra reported “*everyone*” in 63.8 and 79.3 % of respondents respectively, compared to just 22.7 % in Morrumbala. Province-wide, between 2010 and 2014, the proportion of respondents who reported sleeping under a mosquito net in the night prior to survey implementation increased across all populations groups surveyed, with the largest proportional increase seen for the female head-of-household (46.9–83.8 %) and children 0–12 months (48.3–71.2 %).

**Table 3:** Mosquito net utilization in Zambézia Province, Mozambique

	Baseline	Endline
	(n = 3749)	(n = 3892)
Inventory of household mosquito nets		
Missing, n (%)	101 (2.7 %)	219 (5.6 %)
None	35.2 %	35.7 %
Less than the number of beds	23.0 %	35.4 %
One for every bed	38.4 %	25.5 %
More than the number of beds	3.5 %	3.3 %
Who in this household normally sleeps under mosquito nets?		
Missing, n (%)	66 (1.8 %)	205 (5.3 %)
The man of the house	1.7 %	1.3 %
The respondent	3.1 %	0.9 %
The children	10.2 %	10.0 %
Everyone	48.3 %	50.8 %
Others	1.5 %	2.0 %
No one	35.2 %	35.0 %
Head-of-household slept under mosquito net previous night	46.9 %	83.8 %
Received a mosquito net during last pregnancy	34.9 %	54.2 %
If pregnant, slept under mosquito net last night	58.6 %	68.4 %
Child 0–12 months old slept under mosquito net previous night	48.3 %	71.2 %
Child 0–59 months old slept under mosquito net previous night	49.6 %	59.5 %

Continuous variables are reported as weighted estimates of median (interquartile range), with each observation being weighted by the inverse of the household sampling probability

Categorical variables are reported as weighted percentages, with each observation being weighted by the inverse of the household sampling probability

In analysis of just the three oversampled districts, multivariable logistic regression was used to assess changes over time in mosquito net utilization, as well as factors at endline associated with use of a mosquito net in the night prior to survey administration. In comparison to baseline, female heads-of household (OR 7.67, 95 % CI 6.43, 9.16), women pregnant at the time of survey administration (OR 2.17, 95 % CI 1.78, 2.64), and children 0–59 months (OR 3.48, 95 % CI 2.80, 4.33) all had a two to eightfold higher odds of sleeping under a mosquito net the previous night in 2014 (Table 4).

**Table 4:** Logistic regression for mosquito net utilization: endline vs. baseline in the districts of Alto Molócuè, Morrumbala, and Namacurra

	Number in model	OR (95 % CI)	p value
Head-of-household slept under bed net in previous night	4742	7.67 (6.43, 9.16)	<0.001
Received bed net during last pregnancy	3102	2.31 (1.88, 2.85)	<0.001
If pregnant, slept under bed net in previous night	3116	2.17 (1.78, 2.64)	<0.001
Child (0–12 months) slept under bed net in previous night	1064	3.72 (2.56, 5.39)	<0.001
Child (0–59 months) slept under bed net in previous night	3005	3.48 (2.80, 4.33)	<0.001

The odd ratio comparing endline survey with baseline survey ignoring intervention receipt. Effect estimates are from linear regression and represent the average change in the indicator from baseline to endline ignoring intervention receipt

Test of association between survey period and indicators. This does correspond to the odds ratio

Adjusted for: rural/urban; sex of child; maternal education; transportation; household size; traditional healer use in past 12 months

Adjusted for: rural/urban; age of child; sex of child; maternal education; household size; Portuguese speaker

Adjusted for: rural/urban; age; marital status; education; household size; Portuguese speaker

Adjusted for: rural/urban; age; marital status; education; household size; Portuguese speaker; traditional healer use in past 12 months

For female head-of-household respondents, the characteristics at endline associated with mosquito net utilization in the previous night were higher education, understanding Portuguese, larger household size, having electricity in the household, and larger household monthly income (Table 5). Mode of acquisition for mosquito nets, whether all donated or all purchased, was significantly associated with mosquito net utilization. Compared to Alto Molócuè, respondents from Namacurra were more likely (OR 4.51, 95 % CI 3.06–6.65) to report sleeping under a mosquito net the previous night; while respondents from Morrumbala had a 25 % lower odds (OR 0.75; 95 % CI 0.52–1.80, p < 0.001 for district). As travel time to a health facility increased (per 1 h), respondents had 13 % lower odds of sleeping under a mosquito net (OR 0.87; 95 % CI 0.74, 1.01, p = 0.07).

**Table 5:** Determinants of mosquito net use during the previous night among all female and pregnant respondents at endline: districts of Alto Molócuè, Morrumbala, and Namacurra

	Female head-of-household^a^			Pregnant head of household^b^		
	Estimate	(95 % CI)	p value	Estimate	(95 % CI)	p value
Age (per 10 years)	1.09	(0.94, 1.27)	0.25	1.02	(0.73, 1.45)	0.89
Education (6 vs. 0 years)	1.19	(0.84, 1.71)	0.03	1.93	(0.85, 4.36)	0.11
Understands Portuguese	2.10	(1.54, 2.87)	<0.001	1.89	(0.98, 3.65)	0.05
Household size (4 vs. 2 members)	2.03	(1.31, 3.14)	0.01	1.21	(0.93, 1.58)	0.16
District			<0.001			0.002
Alto Molócuè (ref)	1	1		1	1	
Morrumbala	0.75	(0.52, 1.08)		0.91	(0.37, 2.24)	
Namacurra	4.51	(3.06, 6.65)		2.40	(0.91, 6.32)	
Bed nets in house were donated	11.9	(8.89, 15.9)	<0.001	13.36	(7.84, 22.76)	<0.001
Bed nets in house were purchased	20.8	(13.6, 31.9)	<0.001	27.25	(12.79, 58.04)	<0.001
Monthly income (2000 vs. 0 MT)	2.60	(1.74, 3.91)	<0.001	1.64	(0.90, 2.98)	0.08
Travel time to health facility (per 1 h)	0.87	(0.74, 1.01)	0.07	0.92	(0.71, 1.20)	0.55
House has electricity	2.15	(1.03, 4.47)	0.04	1.36	(0.38, 4.81)	0.64
Given a bed net during current pregnancy	–	–	–	2.76	(1.49, 5.11)	0.001

Missing values of predictors were accounted for using multiple imputation

^a^There are 2906 respondents included in this multivariable logistic regression model

^b^There are 790 respondents included in this multivariable logistic regression model

After adjusting for demographic variables, pregnant women had 27 times higher odds of sleeping under a mosquito net if all bed nets were purchased (95 % CI 12.79, 58.04, p < 0.001) and 13 times higher odds if all bed nets were received as a donation (95 % CI 7.84, 22.76, p < 0.001). Additionally, pregnant women had 2.4 times higher odds of sleeping under a bed net if they lived in Namacurra (95 % CI 0.91, 6.32, p = 0.002 for district).

**Fig. 2:**
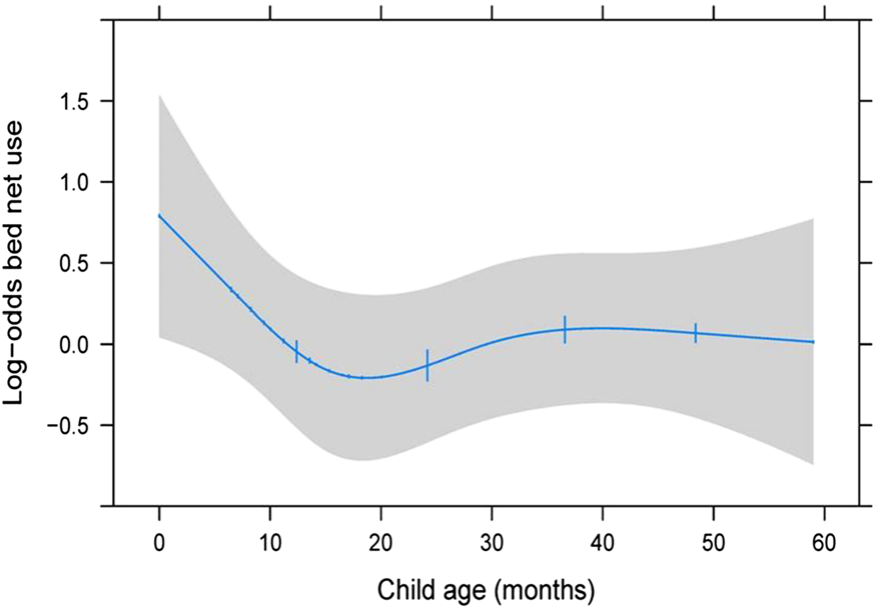
Marginal plot: Log-odds of child mosquito net usage during the night prior to interview by age of child. Adjusted to: maternal education of 2 years, does not understand Portuguese, household size of five, resides in Alto Molócuè, nets were not all donations, nets were not all purchases, monthly income is 500 MZM, travel time is 4 h, no household electricity, and no child fever in the previous 30 days

Similar trends were seen when questioned about mosquito net utilization in children 0–59 months living in the household. Higher maternal education (OR 1.50; 95 % CI 1.09, 2.07, p < 0.001), living in Namacurra (OR 4.18; 95 % CI 2.73, 6.40, p < 0.001), and access to mosquito bed nets were most associated with the child being reported as sleeping under a mosquito net in the previous night (Table 6). Slightly more than half of children 0–59 months slept under a mosquito net in the previous night. When analysed by age, the odds of sleeping under a mosquito net was greatest for newborn children and decreased until about 20 months, such that children aged 24–59 months had similar bed net use (Fig. 2).

**Table 6:** Determinants of mosquito net use during the previous night among children aged 0–59 months at endline: districts of Alto Molócuè, Morrumbala, and Namacurra

	Odds ratio	(95 % CI)	p value
Child age (36 vs. 6 months)	0.75	(0.51, 1.11)	0.16
Mother education (6 vs. 0 years)	1.50	(1.09, 2.07)	<0.001
Understands Portuguese	1.15	(0.85, 1.57)	0.36
Household size (4 vs. 2 members)	1.09	(0.93, 1.29)	0.27
District			<0.001
Alto Molócuè (ref)	1	1	
Morrumbala	0.98	(0.68, 1.41)	
Namacurra	4.18	(2.73, 6.40)	
Mosquito nets in house were donated	3.44	(2.52, 4.71)	<0.001
Mosquito nets in house were purchased	3.06	(1.90, 4.92)	<0.001
Monthly income (2000 vs. 0 MT)	1.63	(1.03, 2.57)	0.10
Travel time to health facility (per 1 h)	1.06	(0.96, 1.18)	0.24
House has electricity	0.83	(0.39, 1.78)	0.64
Child has had fever in the previous 30 days	1.09	(0.83, 1.43)	0.53

There are 2936 children included in this multivariable logistic regression model

Missing values of predictors were accounted for using multiple imputation

## Discussion

Mozambique´s national vector control strategy includes achieving universal access to at least one of the following interventions: (1) living in a household sprayed with insecticide or (2) having access to ITNs [6]. As a public health intervention, ITNs have been shown to have both direct and indirect effects on the dynamics of malaria transmission in a community. When used by high-risk populations such as children under 5 years old and pregnant women, ITNs are known to decrease both overall malaria mortality and all-cause child mortality [25–30]. Additionally, modelling has shown that when a critical mass of at-risk persons are consistently using ITNs, the indirect protective effect at a community level can be greater than the direct effect of individual use [9].

The study examined changes since baseline in ITN use and the factors associated with use of ITNs in Zambézia Province, Mozambique at endline. No survey questions were asked related to indoor residual spraying. The baseline survey was conducted at the end of dry season in August–September and the endline survey was conducted in April–May, directly after the end of rainy season (December 2013–April 2014). The overall household possession (64.3 %) of a least one mosquito net among the study population is consistent with ITN coverage reported in neighboring Tete Province (62.5 %) during a similar household survey conducted at the beginning of rainy season, December 2013 [8]. Furthermore, it is compatible with achieved results reported in prior national surveys and reflects a continuation of the steady rise in Mozambican household ITN coverage over time [3, 5, 31]. Nevertheless, it is still far less than the universal (100 %) coverage recommended by the WHO [1]. Intense efforts are underway to further expand ITN distribution across the country, but it remains unclear as to how to achieve “universal coverage” and how to measure its success or not. Definitions of universal coverage have ranged from distribution of a fixed number of bed nets per household regardless of size, to distribution of one net per every two persons in the household, or finally to having one net per household sleeping space [4, 6]. Per Mozambican national policy as of 2009, each household should have at least one mosquito net for every two persons living there [5]. While these surveys did not directly address the one net per two person policy, results showed that for priority target populations (children <5 years old and pregnant women), the proportion reporting sleeping under a mosquito net in the night prior to survey implementation increased in 2014 as compared to 2010 (Table 3).

Socio-economic factors including higher education, understanding Portuguese, having a higher household income, and living in a house with electricity were positively associated with the female head-of-household sleeping under a mosquito net on the night prior to being interviewed. These findings are consistent with studies in which women and children from households with higher income, positively benefited from both the accessibility and affordability of preventive and curative measures for malaria [32, 33]. Despite this, free distribution of bed nets has been shown to contribute to increased coverage and equity in their use. When commercial sectors are the only option for bed net availability, ownership is limited to the richest families [34]. Distance to a health facility was inversely related with odds of sleeping under a mosquito net in the previous night. Proximity to health facilities, markets, and town centers have been linked in other studies to increased ITN utilization, likely due to improved transportation accessibility and access to mosquito net distribution points [14, 35]. Other studies in Mozambique have found that poorer households were no less likely to own a bed net, just less likely to “buy” a bed net [36]. With this in mind, generalized accessibility through a variety of alternatives becomes an important factor. For rural Zambézia Province, in which 58.2 % of its population is considered multi-dimensionally poor [20], the finding that more bed nets were purchased than received through donations, highlights that current Mozambican strategies to achieve universal ITN coverage through mass donation strategies have not yet reached saturation. In order to maximize household mosquito bed net ownership and use, a combination of both free distribution strategies as well as commercial options will likely be needed.

Current strategies for ITN use in Mozambique call for 80 % coverage in high-risk populations such as children under 5 years old and pregnant women [4, 5]. Results (59.9 and 68.4 % coverage, respectively) still fall considerably short of that target. Like female heads-of-household in general, socio-economic factors were most associated with a pregnant woman sleeping under a bed net, though these trends did not quite reach the same statistical significance (Table 5). The association between higher maternal education and ITN use by children under five years old (OR 1.50, p < 0.001; Table 6) has been noted by other studies. Educated parents are both more likely to come into contact with malaria prevention educational materials and more likely to encourage children to sleep under a net [7, 37, 38].

Reported findings show continued progress in Zambézia Province in terms of household ITN coverage and the proportion of high-risk populations utilizing a bed net in the prior night. However these province-wide estimates obscure variations of progress among and within districts and geographic regions. When looking at only three oversampled districts, Morrumbala performs considerably less in terms of ITN coverage (34 %) and utilization uptake compared to Alto Molócuè and Namacurra. Namacurra outpaced national goals with 89.6 % of households reporting at least one ITN and with 87.6 % of pregnant women and 88.9 % of children under five years old reporting sleeping under a mosquito bed net in the prior evening. Alto Molócuè, has not achieved national targets, yet appears to be following more closely to national trends and on pace for meeting national targets in the near term.

It is important to note that at the time of endline survey implementation, Morrumbala was a district that had not yet benefited from strategies for universal bed net distribution through the Ministry of Health, PMI, or the current Global Fund malaria programme, though it has been covered by indoor residual spraying (IRS) since 2007. In comparison, Alto Molócuè began receiving universal mass ITN campaigns in 2012, and Namacurra in 2013. As of 2014, the only ITNs that were distributed in Morrumbala were done so at antenatal care clinics as part of programmes funded through PMI. All the same, only 24.5 % of pregnant women in Morrumbala reported receiving a mosquito bed net during their last pregnancy, compared to 64 and 74.2 % reported for Alto Molócuè and Namacurra, respectively. Provincial health supply chain systems for commodity distribution are the same whether the commodity is intended for health facility interventions or community related health campaigns. The fact that other districts are currently prioritized ahead of Morrumbala to receive ITN under the universal coverage strategies, likely has resulted in an overall decreased number of free mosquito bed nets available in Morrumbala, including ITNs intended for distribution within antenatal care clinics. Universal distribution of free ITNs is currently planned to begin taking place in Morrumbala in 2017 with Global Fund support.

## Conclusions

Large-scale investments are currently underway to expand both prevention and treatment strategies to combat malaria in Mozambique. Zambézia Province, one of the four identified high malaria prevalence and high population-dense provinces, will continue to be a focus for interventions from programmes such as PMI and the Global Fund. Strategies aimed at maximizing the impact of ITN include “universal” ITN distribution defined as one ITN per every two persons and distribution of ITN to pregnant women at antenatal care clinics. Described here are the province wide disparities in work done to date. Of particular concern is that interventions seem to favor those households of higher socioeconomic status. Intensified efforts and focused attention on the poorest, least educated, and most distant from health services is needed to improve equity of ITN availability and usage. Additionally, while some districts have already surpassed goals in terms of coverage and utilization of ITN, renewed emphasis should be placed on bringing all geographic regions of the province closer to meeting these targets.
